# Cryo-EM-guided subtractive optimization of a novel VCP/p97 inhibitor

**DOI:** 10.1107/S2052252526004604

**Published:** 2026-06-22

**Authors:** Jason Crawford, Ravi Munuganti, Charles Leung, Kriti Singh, Ellen Gates, Xing Zhu, Marcel Bally, Nancy Dos Santos, Maryam Sharifiaghdam, Zeynab Nosrati, Peter Axerio-Cilies, Alison M. Berezuk, Spencer Cholak, Alan Merk, Dale R. Cameron, Sriram Subramaniam

**Affiliations:** a Gandeeva Therapeutics Inc., Burnaby, BC, Canada; b BC Cancer Research Centre, Vancouver, BC, Canada; c Department of Biochemistry and Molecular Biology University of British Columbia Vancouver BC Canada; d Laboratory of Cell Biology National Cancer Institute NIH Bethesda MD USA; e West Coast Chemistry Services, Vancouver, BC, Canada; Max Planck Institute of Biophysics, Germany

**Keywords:** VCP/p97, cryo-EM, small molecule, AML

## Abstract

We report the cryo-EM structure-guided discovery of GND-135, a novel small-mol­ecule inhibitor of the VCP/p97 AAA ATPase that demonstrates efficient inhibition of VCP/p97 in biochemical, cellular, and pharmacokinetic assays and in a tumor efficacy mouse model of acute myeloid leukemia. Our approach overcomes the liability in the clinical-stage com­pound CB-5083 where Phase I studies showed off-target activity of CB-5083 for the enzyme PDE6. From the cryo-EM structural analysis of CB-5083 bound to PDE6 and VCP/p97, we identified critical ligand/protein inter­actions in both proteins and rationally designed a small mol­ecule that retains key inter­actions necessary for VCP/p97 inhibition while eliminating PDE6 off-target activity. We refer to this approach as ‘subtractive optimization’ because we are leveraging our ability to determine both on-target and off-target cryo-EM structures to guide the medicinal chemistry campaign to enable more targeted com­pound design. While this strategy is not possible in all cases, the use of cryo-EM to tune on-site binding while eliminating off-target binding could be a generally applicable strategy for informing mol­ecular design and accelerating small-mol­ecule drug discovery.

## Abstract 2

Our studies show that cryo-EM structure-guided optimization to eliminate the off-target liability of CB-5083 yields a potent and selective inhibitor of the AAA ATPase VCP/p97, a cancer target.

## 1. Introduction

The ATPases Associated with diverse cellular Activities (AAA) ATPase VCP/p97, also known as valosin-containing protein (VCP), serves to regulate protein homeostasis by facilitating the translocation of ubiquitinated proteins from cell membranes or chromatin to the proteasome for degradation (Wang *et al.*, 2020 ▸; Hänzelmann & Schindelin, 2011 ▸;Stach & Freemont, 2017 ▸). In addition to its evolutionarily conserved role in the ubiquitin–proteasome system, VCP/p97 also regulates other key degradation processes, such as autophagy (Ju & Weihl, 2010 ▸; Hill *et al.*, 2021 ▸). Certain cancers, including multiple myeloma (MM) and acute myeloid leukemia (AML), are known for the aberrant overexpression of proteins, resulting in a high dependency on protein folding and homeostasis pathways (Aronson & Davies, 2012 ▸; Nishimura *et al.*, 2019 ▸; Xie *et al.*, 2024 ▸; Ho Zhi Guang *et al.*, 2019 ▸). Thus, these cancer cells could be susceptible to the inhibition of VCP/p97 by causing intra­cellular proteotoxic stress (Roux *et al.*, 2021 ▸; Vekaria *et al.*, 2016 ▸; Le Moigne *et al.*, 2017 ▸). Beyond oncology, VCP/p97 is also known to be an emerging target for neurodegenerative and viral infection indications (Huryn *et al.*, 2020 ▸; Watts *et al.*, 2004 ▸; Johnson *et al.*, 2010 ▸; Das & Dudley, 2021 ▸; Koppers *et al.*, 2012 ▸; Kimonis *et al.*, 2000 ▸; Arita *et al.*, 2012 ▸; Meyer & Weihl, 2014 ▸).

Several classes of small-mol­ecule inhibitors of VCP/p97, including ATP com­petitive, allosteric, and covalent, have been identified and evaluated in preclinical models (Vij *et al.*, 2006 ▸; Huryn *et al.*, 2020 ▸; Anderson *et al.*, 2015 ▸; Zhang *et al.*, 2023 ▸). CB-5083, a potent ATP com­petitive inhibitor of the D2 domain (Zhou *et al.*, 2015 ▸), entered human Phase 1 clinical trials in 2015, but trials were terminated due to side effects that were later traced to strong inter­action with the phospho­diesterase PDE6 (off-target binding) (Leinonen *et al.*, 2021 ▸). A structurally related com­pound with somewhat im­proved selectivity, CB-5339, entered Phase 1 clinical trials in humans for the treatment of hematological malignancies in 2020 (Roux *et al.*, 2021 ▸; Kilgas & Ramadan, 2023 ▸). The com­pound was reported to be well tolerated and demonstrated signs of clinical activity (NCT04402541), but no reports yet exist for progression to Phase 2 trials.

## 2. Experimental strategy

Cryo-EM analysis of the VCP/p97 bound to ADP and ATP has provided insight into the overall hexa­meric organization of the protein (Banerjee *et al.*, 2016 ▸). We chose high-resolution cryogenic electron microscopy (cryo-EM) as an approach to eliminate the liability arising from the off-target effects of these com­pounds. We first determined the cryo-EM structures of CB-5083 bound to native bovine PDE6 (αβγ_2_, full-length, MW ∼210 kDa), which is a surrogate for human PDE6, and to purified VCP/p97 (dodeca­mer, full-length, MW ∼1 MDa), to identify inter­actions at the binding sites that could be selectively modified to prevent PDE6 binding while still retaining potent on-target activity. We then carried out a rational medicinal chemistry hit-to-lead campaign beginning with the development of a novel core that captured many of the inter­actions in the binding site and progressively im­proved selectivity through the synthesis of a series of com­pounds, with optimization at each step confirmed by a primary p97 inhibition assay, with most com­pounds evaluated with secondary assays on a weekly basis, including cryo-EM structures, as well as off-target activities. Using this approach, we identified GND-135 (Crawford *et al.*, 2023 ▸), a potent, on-target, cell-permeable, and orally bioavailable binder with the synthesis of fewer than 60 new com­pounds.

## 3. Results

**Figure 1:**
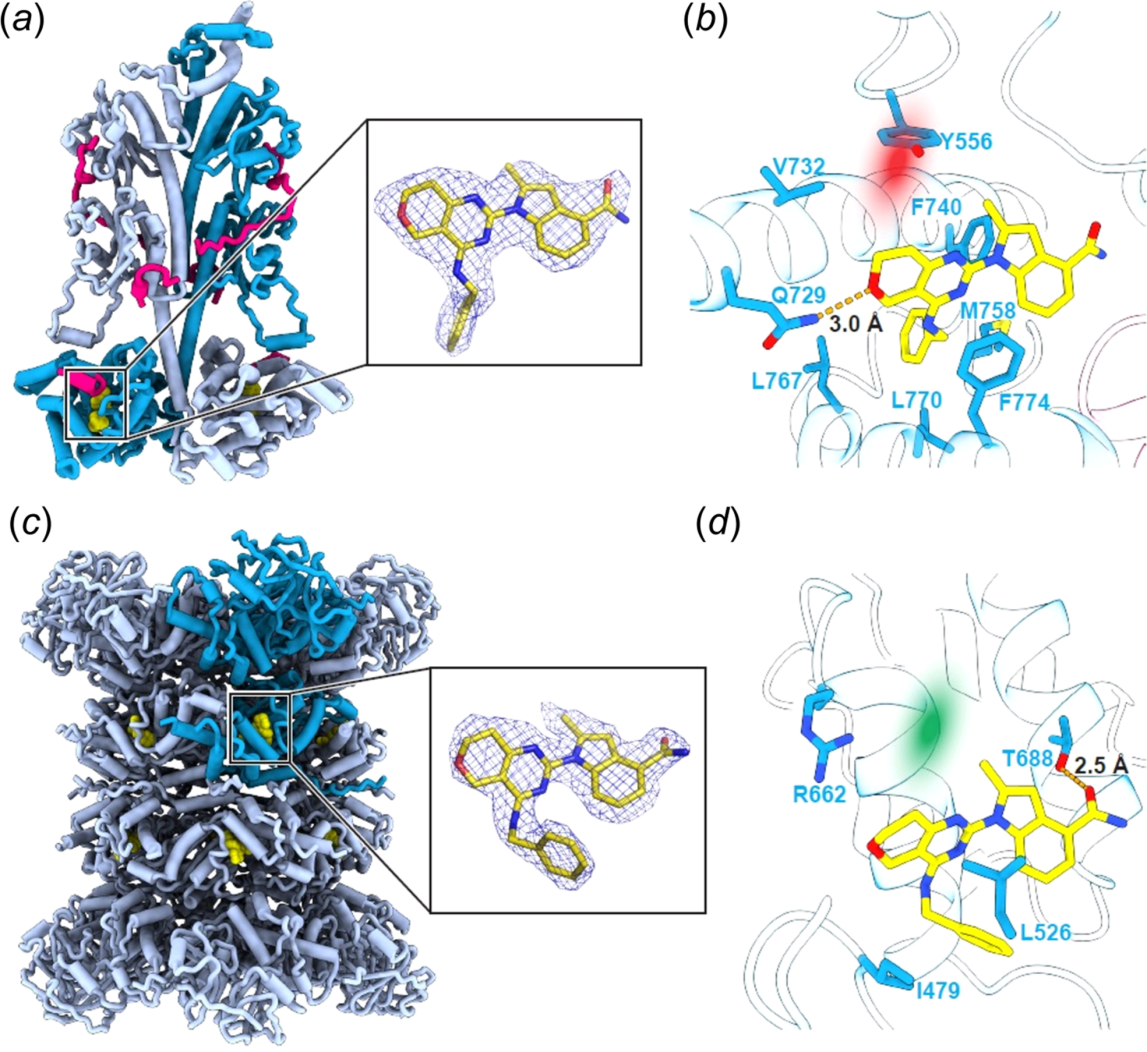
Cryo-EM structures of PDE6 and VCP/p97 in the com­plex with CB-5083. (*a*) The overall structure of PDE6 bound to CB-5083. The PDE6 α, β, and γ subunits are colored light blue, sky blue, and magenta, respectively. CB-5083 is shown in yellow spheres. The inset displays the experimental cryo-EM density for CB-5083 in the map. (*b*) Close-up view of the CB-5083 binding pocket in PDE6. Key inter­acting residues are shown in stick representation, with hy­dro­gen bonds indicated by the dashed orange lines. The transparent red oval indicates a key region of the binding pocket where there are close packing inter­actions between CB-5083 and residues in the PDE6 active site. (*c*) The overall structure of VCP/p97 bound to CB-5083 as we reported previously (PDB ID: 7rli). A single VCP/p97 protomer within the dodeca­mer is highlighted in sky blue. The inset displays the experimental cryo-EM density for CB-5083 in the map. (*d*) Close-up view of the CB-5083 binding pocket in VCP/p97. Key inter­acting residues are highlighted and labeled, with hy­dro­gen bonds indicated by dashed orange lines. In contrast to the close inter­actions seen in the PDE6 binding pocket at the site corresponding to the green oval, there is a cavity observed near the same atoms of CB-5083 in the com­plex with VCP/p97, providing an opportunity to introduce bulkier moieties in this region that would be accommodated in the VCP/p97 pocket but not in that of PDE6.

The 2.72 Å resolution cryo-EM structure of CB-5083 bound to native bovine PDE6 allows visualization of the precise pose and key inter­actions of the com­pound in the binding pocket [Fig. 1 ▸(*a*)]. CB-5083 is anchored to the protein *via* a hy­dro­gen bond (3.0 Å) between the O atom of the di­hydro­pyran ring and the side chain of Q729 [Fig. 1 ▸(*b*)]. F740 establishes an edge-to-face π–π inter­action with the indole ring, and F774 forms an arene–arene inter­action with the pyrimidine ring of the bicyclic core. Additionally, CB-5083 makes strong hydro­phobic contacts with surrounding residues, such as M758, L767, and L770. CB-5083 inhibits PDE6 by com­petitively occupying its catalytic site, blocking cGMP access and hydrolysis to GMP. This results in intra­cellular cGMP accumulation and disrupts signaling. Although originally developed as a p97 inhibitor, CB-5083 binds PDE6 with high affinity, observed in the present cryo-EM structure using conserved hy­dro­gen bonding, π–π stacking, and hydro­phobic contacts, which is consistent with the inhibition mechanisms of other well-characterized PDE inhibitors (Bondarev *et al.*, 2022 ▸). In contrast, CB-5083 bound to VCP/p97 is stabilized by a hy­dro­gen-bond inter­action between its terminal amide and T688, along with nonpolar inter­actions involving the di­hydro­pyran­opyrimidine scaffold, the pendant benzyl­amine group, and nearby residues, such as L526 and I479 [Figs. 1 ▸(*c*) and 1(*d*)].

**Figure 2:**
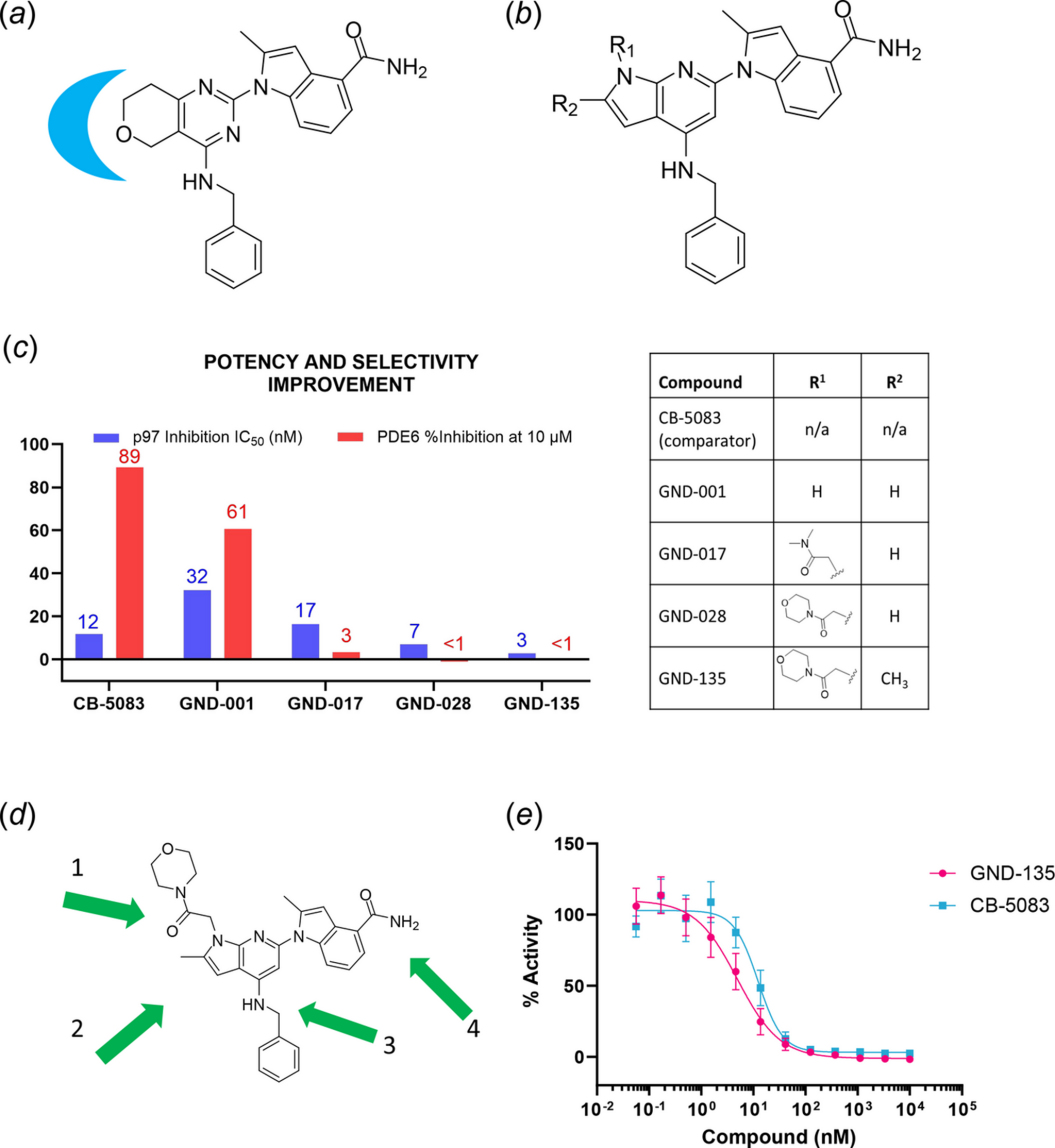
Subtractive optimization strategy to develop a selective VCP/p97 inhibitor. (*a*) The chemical structure of CB-5083. The cyan-colored crescent adjacent to the pyran ring highlights the region that has close packing steric inter­actions in the PDE6 binding site, also highlighted by the red oval in Fig. 1 ▸(*b*). (*b*) The chemical structure of the scaffold used to generate com­pounds that were designed to retain VCP/p97 inhibition but unable to bind to PDE6, highlighting the two sites for functionalization by introducing different *R*^1^ and *R*^2^ groups. (*c*) Compound potency against VCP/p97 ATPase (IC_50_ in n*M*) and PDE6 activity (%Inhibition at 10 µ*M*) for selected com­pounds shown alongside their chemical structures, where *R*^1^ and *R*^2^ refer to the structural sites marked in part (*b*), ultimately resulting in the GND-135 com­pound. IC_50_ values are represented as geomean from independent experiments (*N* ≥ 2) and %Inhibition as the mean of replicates (*n* = 2). (*d*) The chemical structure of GND-135. The green arrows mark the locations of some of the key inter­actions in the binding pocket including (1) the functionalization that sterically precludes PDE6 binding, (2) the removal of a hy­dro­gen-bond acceptor atom from the pyran ring in CB-5083 that contributes to PDE6 binding and (3, 4) sites that maintain strong polar inter­actions with nearby VCP/p97 residues, contributing to selective VCP/p97 inhibition. (*e*) VCP/p97 inhibition dose response curves for CB-5083 and GND-135. Data points are represented as mean ± SD for replicates (*n* = 3).

Comparison of the cryo-EM structures of CB-5083 bound to the two proteins revealed the presence of significantly more steric space around the di­hydro­pyran ring in VCP/p97, which provides an opportunity for mol­ecular extension [Figs. 1 ▸(*b*) and 1(*d*)] and a rational approach to subtractive optimization of on-target activity. There are earlier structures reported for CB-5083 bound to full-length VCP by cryo-EM (Caffrey *et al.*, 2021 ▸) and to the D1D2 domain by X-ray crystallography (Tang *et al.*, 2019 ▸); we used the cryo-EM structure in our com­pound design strategy. We first noticed that some of the key inter­actions that are important in PDE6 binding involve a region of CB-5083 around the di­hydro­pyran ring [Fig. 2 ▸(*a*)], where there is additional empty space when CB-5083 is bound to VCP/p97. This immediately suggested a strategy where we replace the di­hydro­pyran ring and systematically add substituents near the di­hydro­pyran ring (or isosteric equivalent) that would sterically block binding to PDE6 but could be accommodated in VCP/p97. Our strategy for rational ‘sub­tractive optimization’ to eliminate PDE6 activity was based on adding steric bulk near the di­hydro­pyran ring [Fig. 2 ▸(*b*)], while maintaining other key inter­actions, such as the pendant aryl group and the terminal amide group that drives potency at the solvent-exposed portion.

To systematically evaluate the effects of making changes at these sites and to overcome the off-target reactivity, we used a combination of biochemical assays to measure VCP/p97 ATPase activity, a cellular CHOP luciferase-reporter assay that measures induction in response to unfolded protein response (UPR) (Oyadomari & Mori, 2004 ▸), and cryo-EM at each step to guide the systematic design of mol­ecules with the goal of maintaining VCP/p97 biochemical and cellular activity while removing PDE6 cross-reactivity.

**Figure 3:**
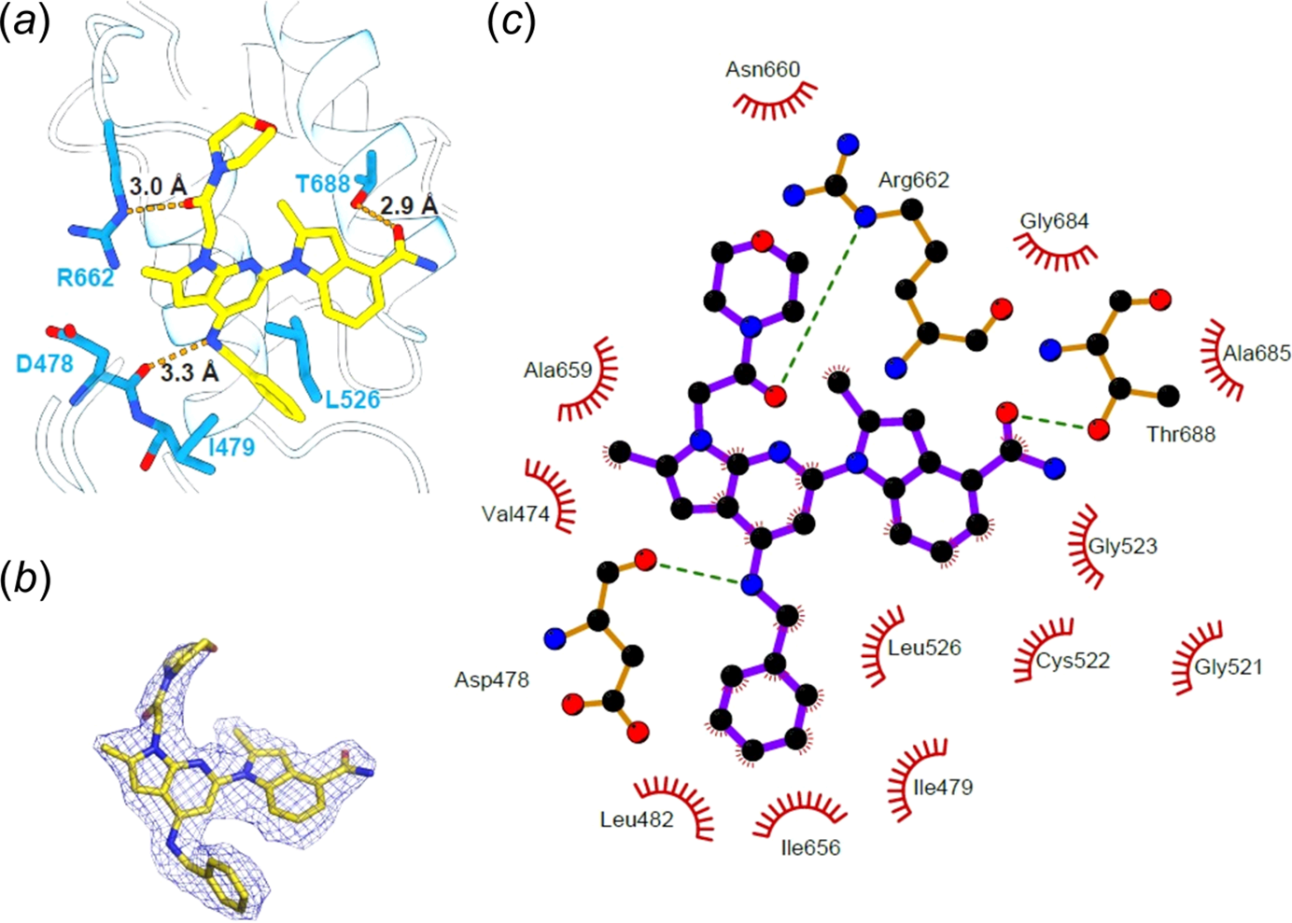
GND-135 eliminates PDE6 cross-reactivity while maintaining VCP/p97 potency. (*a*) Detailed view of the binding pose of GND-135 in the VCP/p97 binding site. Key inter­acting residues are shown in stick representation, with hy­dro­gen bonds indicated by the dashed orange lines. (*b*) The binding pose of GND-135 in the binding pocket is unambiguously visualized as indicated by the experimental cryo-EM density in the map. (*c*) Ligplot showing the specific residues involved in the binding of GND-135 in the VCP/p97 binding pocket. Hydrogen bonds are indicated by green dashed lines.

Initial modification of the bifunctional aromatic core to a 7-aza­indole core yielded GND-001 [Fig. 2 ▸(*c*) and Table 1 in the supporting information]. Although a slight reduction in biochemical and cellular potency was observed, PDE6 inhibition was reduced com­pared to CB-5083 (61% *versus* 89% PDE6 inhibition at 10 µ*M*). The 7-aza­indole core supported our docking predictions where the distinct heteroatom position avoided the critical hy­dro­gen-bond inter­action with Gln729 of PDE6 and the indole N atom allowed for the attachment of additional functionalities to further preclude PDE6 binding. Functionalizing the indole core at the *R*1 position led eventually to GND-017 and GND-028 with im­proved biochemical and cellular potency, with virtual elimination of PDE6 cross-reactivity (<3% PDE6 inhibition at 10 µ*M*) com­pared to GND-001 [Fig. 2 ▸(*c*) and Table 1 in the supporting information]. The bulky acetyl morpholine *R*1 group maintains inter­actions with VCP/p97 polar residues R662 and K663, while sterically clashing with the smaller PDE6 pocket. With the PDE6 cross-reactivity resolved with GND-028, efforts were focused on im­provements in bio­chemi­cal and cellular potency *via* modifications in other parts of the mol­ecule. Attempts to enhance potency through modifications of the indole and benzyl­amine moieties were largely unsuccessful, but methyl­ation of GND-028 produced GND-135 [Fig. 2 ▸(*d*)] with approximately twofold im­prove­ment in biochemical and cellular potency and no effect on PDE6 (<1% PDE6 inhibition at 10 µ*M*) com­pared to GND-028 (Table 1 in the supporting information). The pen­dant benzyl­amine group shared by both CB-5083 and GND-135 maintains inter­actions with a hydro­phobic pocket in VCP/p97 and a key polar inter­action with the D478 backbone (Fig. 3 ▸). The significance of the acetyl morpholine group introduced in GND-135 is that it creates additional polar inter­actions with R662, which are likely the main determinants for increased VCP/p97 biochemical potency and loss of PDE6 cross-reactivity com­pared to CB-5083 [Fig. 2 ▸(*e*)].

**Figure 4:**
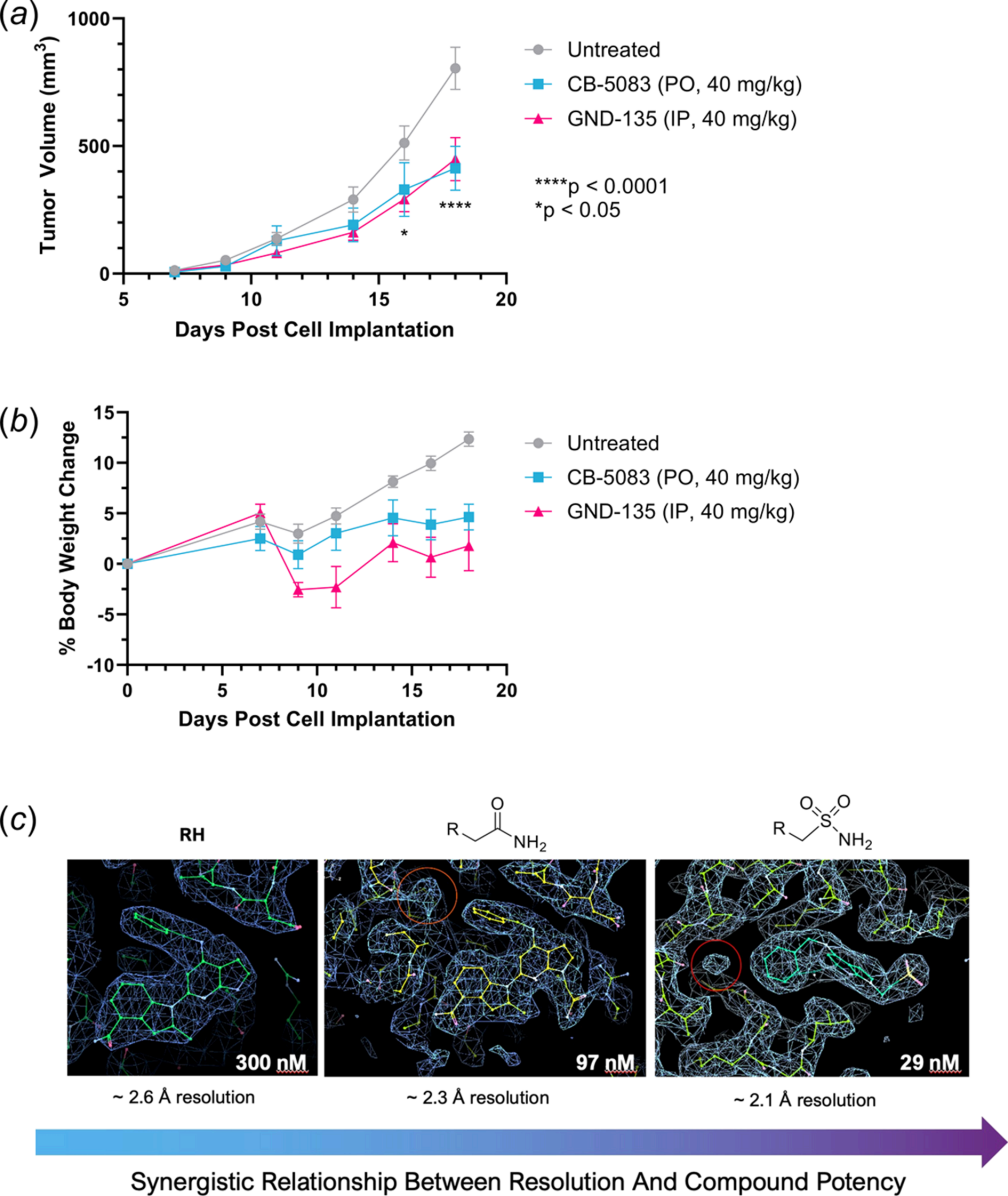
GND-135 has com­parable *in vivo* efficacy com­pared to CB-5083. (*a*) *In vivo* efficacy of GND-135 *versus* CB-5083 in the U937 CDX model. Tumor growth *versus* days post cell implantation for untreated and treated groups. CB-5083 was administered at 40 mg/kg QD, PO, and GND-135 was administered at 40 mg/kg QD, IP. (*b*) %Body weight change *versus* days post cell implantation. Data are represented as mean ± SEM. (*c*) Synergistic relation between cryo-EM map resolution and com­pound potency. As the potency of the com­pound is im­proved with the progression from an H atom to an amide moiety to a sulfonamide moiety, there are more inter­actions in the binding pocket. In turn, this likely results in reduced flexibility of the com­pound. Because VCP/p97 is a dynamic allosteric protein where active site inhibitors bound to D2 restrict conformational flexibility, an increase in local resolution at the binding site driven by more inter­actions is also partially reflected in the resolution of the overall density map.

We next evaluated cellular potencies, absorption, distribution, metabolism, and excretion (ADME) and pharmacokinetic (PK) properties of GND-135 (Tables 2–4 in the sup­porting information). Our experiments indicate that both GND-135 and CB-5083 show com­parable cellular potencies in cell proliferation assays tested in multiple myeloma RPMI-8226 and acute myeloid leukemia U937 cell lines (Table 2 in the supporting information), which are cell models selected for their higher sensitivity to VCP/p97 inhibition. Initial PK experiments with intra­venous (IV) administration showed that the plasma half-life was deemed suitable for daily ad­mini­stration (Table 4 in the supporting information). The com­pound was tolerated at a 40 mg/kg dose with intra­peri­toneal (IP) administration, so we proceeded to the evaluation of the efficacy of GND-135 *versus* CB-5083 in a U937 mouse model of AML. CB-5083 has established *in vivo* and clinical activity, and is used as a com­parative control. In this study, where CB-5083 and GND-135 were given at 40 mg/kg QD (oral administration and IP, respectively), both com­pounds demonstrated equivalent efficacy, which was statistically differentiated from untreated mice [Fig. 4 ▸(*a*)]. GND-135 was administered by IP delivery because our goal was to reach levels of drug in the tumor similar to that achieved with CB-5083 with oral delivery and we knew the oral bioavailability of GND-135 in mice was 14%, *versus* a reported 41% for CB-5083 (Zhou *et al.*, 2015 ▸). No significant change in body weight was found in treated *versus* untreated groups [Fig. 4 ▸(*b*)].

## 4. Discussion

As discussed above, a primary role of VCP/p97 is to recognize ubiquitinated proteins and facilitate translocation to the pro­teasome for degradation (Kloppsteck *et al.*, 2012 ▸). The inhibition of VCP/p97, particularly in cells subject to the aberrant overexpression of proteins, should therefore lead to the excessive accumulation of ubiquitinated proteins and consequent proteotoxic stress (Vekaria *et al.*, 2016 ▸). Applications in oncology, where mutational overexpression of proteins are common, has been the logical primary initial clinical application of the inhibitors. For example, CB-5083 was evaluated preclinically in acute myeloid leukemia (AML) cell lines. Applications in other disease areas may also be valuable. Missense mutations in VCP/p97, which increase ATPase activity, have been implicated in neurodegenerative conditions, such as inclusion body myopathy with early onset Paget disease and frontotemporal dementia (IBMPFD), and familial amyotrophic lateral sclerosis (ALS) (Ritson *et al.*, 2010 ▸; Meyer & Weihl, 2014 ▸; Dec *et al.*, 2014 ▸). With well-tolerated and mechanistically selective tool com­pounds, it may be possible to further explore the links between VCP/p97 inhibition and disease phenotype progression.

Our studies show that cryo-EM structure-guided optimization to eliminate the off-target liability of CB-5083 yields a potent and selective inhibitor of VCP/p97. An important aspect of our strategy is that com­pound design is the synergistic relation between com­pound activity and resolution of the cryo-EM structure at the binding site [Fig. 4 ▸(*c*)]. Increasing activity results in tighter binding of the lead com­pounds, which in turn im­proves the local resolution, allowing clearer delineation of water mol­ecules and hy­dro­gen bonds. These effects occur at the side-chain and com­pound functional group level, with observation of increased ordering (and therefore resolution) of specific groups. Thus, with the synthesis of each new com­pound set, determination of the structures of more potent com­pounds allowed us to design com­pounds with a further likelihood of im­proved activity, which, in turn, provided more specific guidance for the atomic positions where we made the changes for the next round of design. Improved resolution can of course also result from im­proved structural stabilization of the protein. The im­proved visualization of the binding inter­actions enables more rational strategies for im­proving the overall potency, which is determined by a number of other parameters, such as membrane permeability and metabolic stability. We propose that the combination of specifically subtracting off-target atomic inter­actions, combined with the synergy between potency and resolution, could be a general strategy for accelerated structure-guided drug discovery.

## 5. Methods

### 5.1. Compound synthesis

Details of the synthesis of com­pounds can be found in the supporting information. CB-5083 was purchased from Med­ChemExpress (HY-12861).

### 5.2. Protein expression and purification

Full-length VCP/p97 with N-terminal His-tag was over-expressed in BL21DE3 (ThermoFisher). Cell pellets were resuspended in Lysis buffer [100 m*M* Tris pH 8, 500 m*M* NaCl, 1 m*M* β-mercapto­ethanol (BME)] and sonicated. Following centrifugation, cell lysate was purified on a HisPur column (Thermo) and eluted with Elution buffer (25 m*M* Tris pH 8, 150 m*M* NaCl, 1 m*M* MgCl_2_, 300 m*M* imidazole and 1 m*M* BME). Elution fractions were pooled and dialyzed overnight at 4 °C in Dialysis buffer [25 m*M* Tris pH 8, 150 m*M* NaCl, 1 m*M* MgCl_2_, 1 m*M* BME, 200 U Apyrase (Sigma)]. The dialyzed protein was then purified using size-exclusion chromatography with a Superose 6 10/300 GL column (Cytiva) in SEC buffer [25 m*M* Tris pH 8, 150 m*M* NaCl, 1 m*M* MgCl_2_, 1 m*M* tris­(2-carb­oxy­eth­yl)phosphine (TCEP)]. Fractions cor­responding to hexa­meric VCP/p97 (assessed by native PAGE or Refeyn mass photometer) were then pooled and stored at −80 °C for subsequent studies. Purification of PDE6 was carried out as described by Wensel *et al.* (2005 ▸) and concentrated to 1.5 mg ml^−1^ in 20 m*M* sodium phosphate buffer at pH 7.5, 150 m*M* NaCl, 2m*M* di­thio­threitol (DTT).

### 5.3. p97 ATPase assay

The effect of com­pound inhibition on VCP/p97 ATPase activity was evaluated using the ADP-Glo assay (Promega). The ATPase assay was performed in 384-well white low-volume plates (Corning). The 10 µl reaction volumes con­sisted of 110 n*M* VCP/p97 preincubated with com­pound in assay buffer (40 m*M* Tris pH 7.5, 20 m*M* MgCl_2_, 0.1 mg ml^−1^ BSA) for 30 min at room tem­per­a­ture prior to addition of 50 µ*M* ATP. Compounds were prepared from dimethyl sul­foxide (DMSO) stocks to a final DMSO concentration of 0.1 or 0.5%. After 1 h incubation at room tem­per­a­ture, conversion of ATP to ADP was detected according to Promega ADP-Glo assay protocol. Luminescence was measured using a Cytation5 microplate reader (Agilent). Each condition was performed in triplicate and IC_50_ values were determined by fitting to a 4-parameter Hill equation using *CDD Vault* (Burlingame, CA; https://www.collaborativedrug.com) or *Graphpad Prism* (Boston, Massachusetts USA; https://www.graphpad.com/).

### 5.4. PDE6 assay

The PDE6 activity assay was performed at Eurofins Panlabs (Cat. No. 156100). Briefly, bovine retinal rod purified PDE6 was pre-incubated with 1 or 10 µ*M* com­pound at 1% DMSO followed by addition of 100 µ*M* [^3^H]cGMP + cGMP to initiate the reaction. Activity was measured by qu­anti­fying the formation of [^3^H]guanosine.

### 5.5. Cryo-electron microscopy

10 mg ml^−1^ frozen aliquots of purified VCP/p97 were thawed and subsequently centrifuged at 12000 × *g* for 10 min. The resulting supernatant was diluted to a concentration of 3 mg ml^−1^ in 25 m*M* Tris-HCl pH 7.5, 30 m*M* NaCl, 10 m*M* MgCl_2_, 1 m*M* TCEP. Compounds dissolved in 0.1% DMSO were added to a final concentration of 200 µ*M*. Protein–com­pound mixtures (1.8 µl) were then applied to glow dis­charged (15 mA, 30 s) Qu­anti­foil R1.2/1.3 200 mesh copper holey carbon grids. Grids were blotted (blot force −10, wait time 0 s) for 12 s at 10 °C in 100% humidity and plunge-frozen in liquid ethane using a Vitrobot Mark IV (Thermo Fisher Scientific) plunge-freezing device. PDE6+CB-5083 grids were made similarly using a 1.5 mg ml^−1^ concentration of PDE6. VCP/p97 com­plexes were imaged using a 300 kV Titan Krios transmission electron microscope (ThermoFisher Scientific) equipped with a Falcon4 direct electron detector in electron event representation (EER) mode. Movies were collected at 165000× magnification (physical pixel size 0.73 Å) over a defocus range from −0.5 to −3 µm, with a total accumulated dose of 40 e^−^ Å^−2^, using *EPU* automated acquisition software (Thermo Fisher Scientific). The PDE6+CB-5083 com­plex was imaged using a 300 kV Titan Krios transmission electron microscope (Thermo Fisher Scientific) equipped with a K3 direct electron detector (Gatan Inc.) operated in super-resolution mode. Movies were collected at a physical pixel size of 0.5 Å over a defocus range from −0.5 to −3 µm, with a total accumulated dose of 68 e^−^ Å^−2^ using Gatan software.

All data processing was carried out in *CryoSPARC* (Version 4.6.2; Punjani *et al.*, 2017 ▸). Motion correction and CTF estimation were performed in patch mode, followed by blob-based particle picking, extraction, and streaming 2D classification within *CryoSPARC* Live sessions. High-quality particles selected from the streaming 2D classification were used for *ab initio* reconstruction to generate initial maps for 3D classification. For the PDE6+CB-5083 dataset, particles from the highest-resolution 3D class were used for final 3D refinement. For the VCP/p97+GND-135 dataset, three rounds of 3D classification were performed to obtain the final particle stack. The final 3D refinement included per-particle CTF estimation and aberration correction, with D6 symmetry applied. Initial models for structure building were derived from PDB entries 3cf3 (Davies *et al.*, 2008 ▸) (VCP/p97) and 6mzb (Gulati *et al.*, 2019 ▸) (pde6). Inhibitor com­pounds were manually docked into the active sites. Model adjustments were performed in *COOT* (Emsley *et al.*, 2010 ▸), followed by iterative rounds of refinement using *COOT* and *Phenix* (Lieb­schner *et al.*, 2019 ▸). Details of the single-particle cryo-EM workflows and validation are provided in Figs. 1 ▸ and 2 ▸ and Table 5, respectively, in the supporting information.

### 5.6. Cellular CHOP reporter assay

Mia-Paca2 cells stably expressing a luciferase reporter driven by a CHOP-dependent promoter (Signosis) were maintained in DMEM media supplemented with 10% FBS and 2.5% horse serum (Gibco) at 37 °C, 5% CO_2_. Cells were plated at 6500 cells/well in 384-well white TC-treated plates (Nunc). After 24 h incubation, cells were treated with vehicle (DMSO) or com­pounds at a final DMSO concentration of 0.1% and a total volume of 40 µl. After 8 h incubation with com­pounds, 40 µl of Bright-Glo reagent (Promega) was added and the plates were incubated for 2 min with orbital shaking. Luminescence was measured using a Cytation5 microplate reader (Agilent). Each condition was performed in triplicate and EC_50_ values were determined with a 4-parameter Hill equation using *CDD Vault* or *GraphPad Prism*.

### 5.7. Cell viability assay

RPMI-8226 and U937 cells (ATCC) were maintained in RPMI-1640 media (ATCC) supplemented with 10% FBS (Gibco) at 37 °C, 5% CO_2_. Cells were plated at 6500 cells/well in 384-well white TC-treated plates (Nunc). After 24 h incubation, cells were treated with vehicle (DMSO) or com­pounds at a final DMSO concentration of 0.1% and a total volume of 40 µl. After 24 or 72 h incubation for RPMI-8226 or U937 cells, respectively, 40 µl of CellTiter-Glo 2.0 reagent (Pro­mega) was added and the plates were placed on an orbital shaker for 10 min. Luminescence was measured using a Cytation5 microplate reader (Agilent). Each condition was performed in triplicate and IC_50_ values were determined with a 4-parameter Hill equation using *CDD Vault* or *GraphPad Prism*.

### 5.8. Microsomal stability assay

Human and mouse liver microsomal stability assays were performed at Wu Xi AppTec. Briefly, 0.5 mg ml^−1^ microsome was incubated with 1 m*M* com­pound and 1 m*M* NADPH at 37 °C. Samples at various time points up to 60 min were quenched and analyzed using LC-MS/MS. Data were fitted with first-order kinetics to calculate parameters.

### 5.9. Pharmacokinetics

Pharmacokinetic analysis of com­pounds was performed at Wu Xi AppTec in collaboration with X-Chem (Intellsyn). Com­pounds were formulated in 10% *N*-methyl­pyrrolidinone (NMP), 10% solutol, and 80% water to a concentration of 0.9 mg ml^−1^. The com­pounds were administered orally (PO) at 9 mg/kg or intra­venously (IV) at 3 mg/kg to female BALB/c nude mice. Plasma com­pound concentrations were determined by LC/MS analysis on a Shimadzu LC-20AD HPLC using an AB Sciex API 5500 MS detector with terfenadine (5 ng ml^−1^) as an inter­nal control.

### 5.10. *In vivo* efficacy model

All animal research conducted was reviewed and approved by the Institutional Animal Care Committee (IACC) at UBC. Housing and use of animals were performed in accordance with the Canadian Council on Animal Care Guidelines. Female NRG mice aged 8–10 weeks and weighing 18–27 g were obtained from BCCRC.

The tolerability of a 40 mg/kg IP dose of GND-135 was established in female NRG mice with a vehicle formulation control [7.5% NMP, 22.5% PEG400, 20% solutol, 50% saline (0.9% NaCl)].

To determine efficacy, U937 cancer cell line xenografts were established by implanting 2 × 10^6^ cells subcutaneously in female NRG mice. Animal randomization by body weight (8 per group) and dose administration was initiated on day 7 post transplantation. CB-5083 [0.5% methyl cellulose in water (Le Moigne *et al.*, 2017 ▸)] was administered by oral gavage and GND-135 (7.5% NMP, 22.5% PEG400, 20% solutol, 50% saline) was administered intra­peritoneally. Tumor volume and body weights were measured on days indicated. The study was terminated once tumor volumes reached a maximum of 800–1000 mm^3^. Animals were anesthetized with isoflurane, and then with CO_2_ delivered at a flow rate of 4–5 l min^−1^ to a 2.3 l chamber (approximately 200% chamber volume/per minute). The work reported is in accordance with ARRIVE guidelines.

### 5.11. Statistical information

Qu­anti­fication and statistical analysis information can be found in the figure legends associated with the data. For the *in vivo* efficacy model, statistical analysis was performed using Two-way ANOVA with Tukey’s post-hoc test com­paring treated *versus* untreated groups (*n* = 8) using *GraphPad Prism*.

## Supplementary Material

Supplementary figures, tables and methods. DOI: 10.1107/S2052252526004604/kf5008sup1.pdf

PDB reference: cryo-EM structure of bovine phosphodiesterase 6 bound to CB-5083, 9ohm

PDB reference: cryo-EM structure of human p97/VCP bound to inhibitor GND-135, 9ohn

EMDB reference: cryo-EM structure of bovine phosphodiesterase 6 bound to CB-5083, EMD-70500

EMDB reference: cryo-EM structure of human p97/VCP bound to inhibitor GND-135, EMD-70501

## Data Availability

Density maps and fitted coordinates have been made available at the Electron Microscopy Data Bank (EMDB) and Protein Data Bank (PDB), respectively (CB-5083 bound to PDE6+CB-5083: EMD-70500 and 9ohm; VCP/p97+GND-135: EMD-70501 and 9ohn).
