# Supplementary material for: Cryo-EM-guided subtractive optimization of a novel VCP/p97 inhibitor
Source: IUCrJ. 2026 Jun 22;13(Pt 4):364–72. doi: 10.1107/S2052252526004604 (PMC13324599; doi:10.1107/S2052252526004604)
Supplement: Supplementary file 1 [file m-13-00364-sup1.pdf]

IUCrJ

Volume 13 (2026)

Supporting information for article:

Cryo-EM-guided subtractive optimization of a novel VCP/p97 inhibitor

Jason Crawford, Ravi Munuganti, Charles Leung, Kriti Singh, Ellen Gates, Xing Zhu, Marcel Bally, Nancy Dos Santos, Maryam Sharifiaghdam, Zeynab Nosrati, Peter Axerio-Cilies, Alison M. Berezuk, Spencer Cholak, Alan Merk, Dale R. Cameron and Sriram Subramaniam

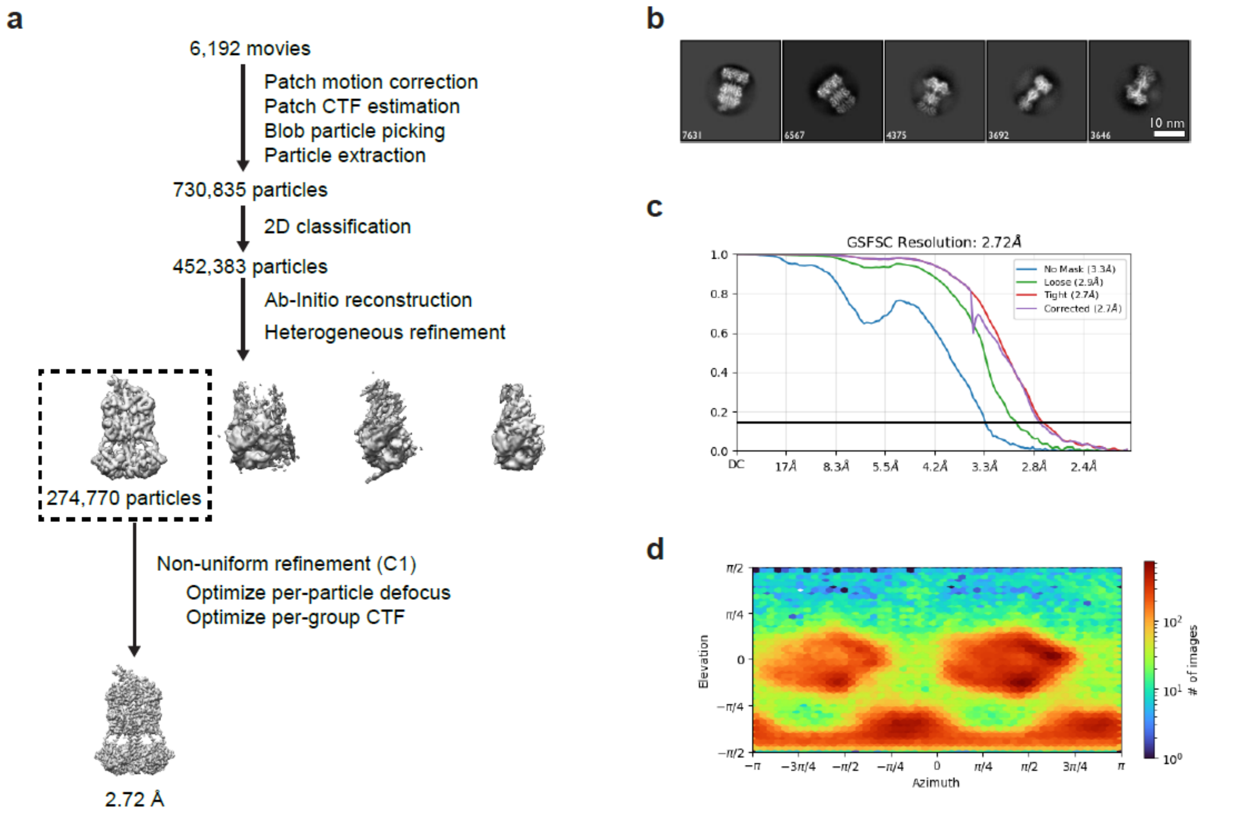

Supplementary Fig. 1. Cryo-EM data processing workflow for the PDE6+CB-5083 complex. (*a*) Data processing flowchart for the PDE6+CB-5083 complex. (*b*) Representative classes from 2D classification. (*c*) Fourier shell correlation (FSC) plot for final refinement. (*d*) Plot of viewing direction distribution.

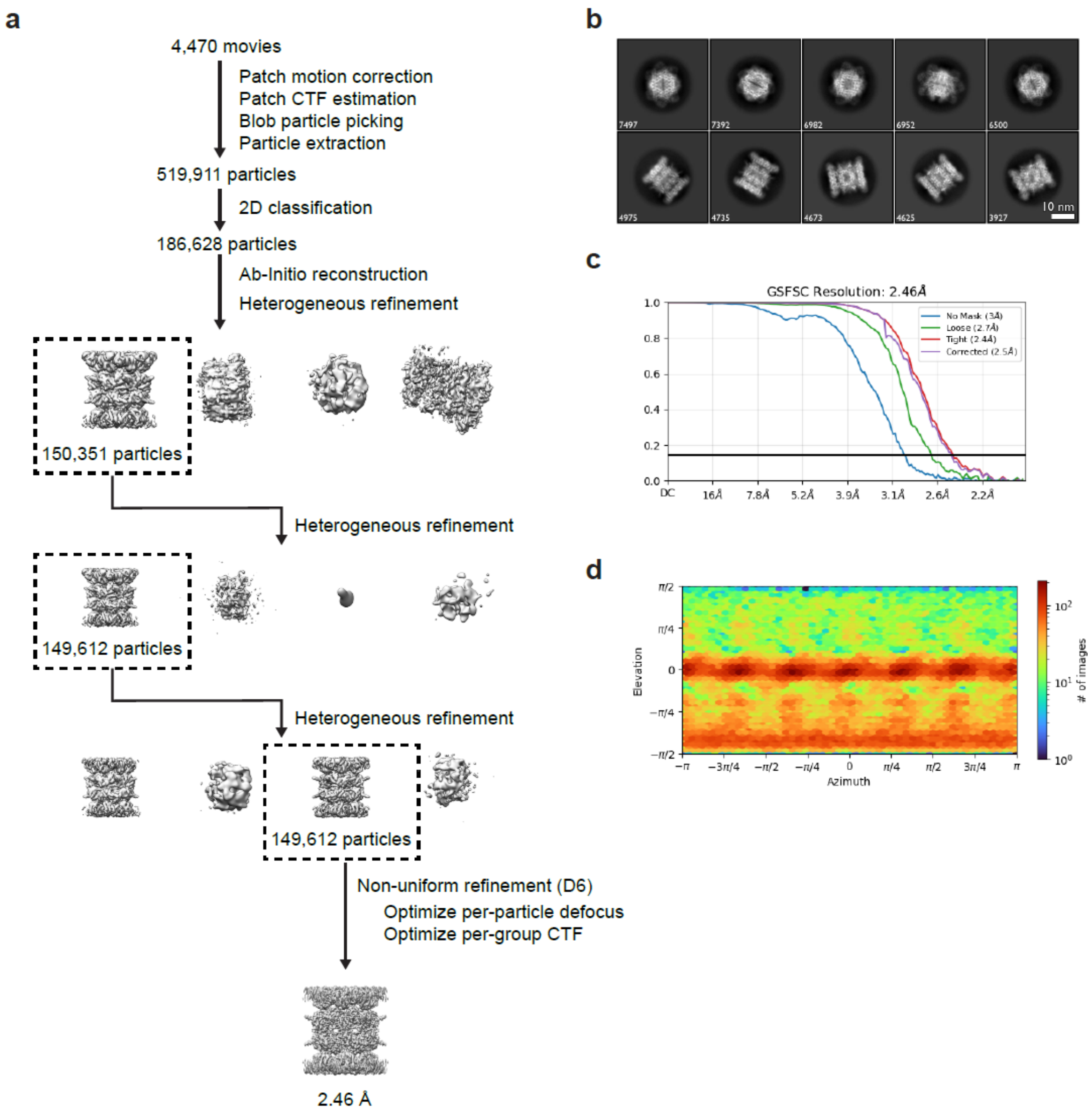

Supplementary Fig. 2. Cryo-EM data processing workflow for the VCP/p97+GND-135 complex. (*a*) Data processing flowchart for the VCP/p97+GND-135 complex. (*b*) Representative classes from 2D classification. (*c*) Fourier shell correlation (FSC) plot for final refinement. (*d*) Plot of viewing direction distribution.

Supplementary Table 1. Comparison of experimental activities of CB-5083 and VCP/p97 inhibitors developed. N = number of independent experiments, n = number of replicates in single experiment.

Compound	VCP/p97 Inhibition IC_{50}, Geomean (μM)	VCP/p97 Inhibition IC_{50}, Standard Deviation (x/÷)	VCP/p97 Inhibition IC_{50}, N	Cellular CHOP EC_{50}, Geomean (μM)	Cellular CHOP EC_{50}, Standard Deviation (x/÷)	Cellular CHOP EC_{50}, N	% of PDE6 Inhibition at 10 μM, Mean	% of PDE6 Inhibition at 10 μM, Standard Deviation (±)	% of PDE6 Inhibition at 10 μM, n
CB-5083	0.012	1.7	28	0.4	1.3	14	89	0.1	2
GND-001	0.032	2.3	2	4	1.2	2	61	6.9	2
GND-017	0.017	3.2	3	2.1	1.3	2	3	1.6	2
GND-028	0.007	7.9	8	1.6	1.2	2	<1	2.5	2
GND-135	0.003	2.3	4	0.8	1.1	2	<1	7.0	2

Supplementary Table 2. Cell viability potencies of GND-135 and CB-5083. N = number of independent experiments.

Compound	RPMI8226 Cell Viability IC_{50}, Geomean (μM)	RPMI8226 Cell Viability IC_{50}, Standard Deviation (x/÷)	RPMI8226 Cell Viability IC_{50}, N	U937 Cell Viability IC_{50}, Geomean (μM)	U937 Cell Viability IC_{50}, Standard Deviation (x/÷)	U937 Cell Viability IC_{50}, N
CB-5083	0.3	1.5	13	0.3	2.1	3
GND-135	0.6	1.4	3	0.7	1.6	3

Supplementary Table 3: ADME properties of GND-135 and CB-5083 (Zhou *et al.*, 2015) as reported in literature.

Assay	CB-5083	GND-135
Microsomal stability ($T_{1/2}$ min; mouse, human)	102, n/a	14, 56

%F in mice	41%	14%

Supplementary Table 4. Mouse IV PK properties of GND-135 and CB-5083 (Zhou *et al.*, 2015) as reported in literature.

#	$T_{1/2}$ (hr)	AUC_{inf} (hr)	Vss (L/kg)	CL(mL/min/kg)
CB-5083	2.83	8.42	0.42	5.9
GND-135	6.63	6.21	1.04	15.9

Supplementary Table 5. Cryo-EM experiments on PDE6+CB-5083 and VCP/p97+GND-135.

	PDE6+CB-5083	VCP/p97+GND-135
	EMDB EMD-70500	EMDB EMD-70501
	PDB ID 9OHM	PDB ID 9OHN
**Data collection**		
Microscope	Titan Krios	Titan Krios
Detector	K3	Falcon4
Voltage (kV)	300	300
Nominal magnification	190,000	165,000
Defocus range (μm)	-3.0 to -0.5	-3.0 to -0.5
Physical pixel (Å)	0.5	0.73
Electron dose ($e^{-}/Å^2$)	68	40
Format of movies	Tiff	EER
Number of movies	6,192	4,470
**Data processing**		
Number of fractions	40	40
Number of extracted particles	730,835	519,911
Number of particles for final map	274,770	149,612
Symmetry imposed	C1	D6
Resolution (Å)	2.72	2.46
FSC threshold	0.143	0.143
**Refinement**		
Initial model used	6MZB	3CF3
Map sharpening B-factor ($Å^2$)	77.0	78.0
Composition (#)		
Atoms	14,219	51,708
Residues	1,734	6,528

Ligands	ZN:2; MG:2; PCG:2; CB-5083:2	ADP:12; GND-135:12
B-factor ($Å^2$)		
Protein (min/max/mean)	24.17/261.26/104.49	45.98/220.49/104.44
Ligand (min/max/mean)	73.57/113.41/93.32	47.75/119.10/79.83
Bonds (RMSD)		
Length (Å) (# > 4σ)	0.003 (0)	0.003 (0)
Angles (°) (# > 4σ)	0.516 (0)	0.611 (0)
CC_mask	0.86	0.83
**Validation**		
Ramachandran plot		
Residues favored (%)	97.15	96.64
Residues disallowed (%)	0.00	0.00
Rotamer outliers (%)	1.16	0.00
Clash score	3.09	5.84
MolProbity score	1.30	1.53

Supplementary Methods: Synthesis of compounds

All temperatures are in degrees Celsius (°C) and are uncorrected. Reagent grade chemicals and anhydrous solvents were purchased from commercial sources and unless otherwise mentioned, were used without further purification. The names of the products were determined using the naming software included in the ChemDraw Professional – PerkinElmer Informatics Desktop Software. Silica gel chromatography was performed on Teledyne Isco instruments using pre-packaged disposable SiO_2 stationary phase columns with eluent flow-rate ranges of 15 to 200 mL/min, UV detection (254 and 280 nm). Reverse phase purification was carried out using C18 columns, UV detection (214 and 254 nm).

The analytical HPLC chromatograms were performed using an Agilent 1100/1200 series instrument with DAD detector (190 nm to 300 nm) and a thermostatted column compartment set to 35 ^oC. The mass spectra were recorded with a Waters SQD detector with the desolvation temperature set to 500 ^oC, the source temperature set to 150 ^oC and the desolvation gas flow set to 1000 L/hr. The mass spectrometer was equipped with an electrospray ion source (ESI) operated in a positive, or negative, ion mode and was set to scan between m/z 100-750 with a scan time of 0.2s. Products and intermediates were analyzed by HPLC/MS on one of 4 methods (A05/A50/B05/B50). Method A05 is a 3.50 minute method using a Kinetex EVO C18 (5 μM, 4.6 x 50 mm) column, a flow rate of 2.20 mL/min, and a low pH buffer gradient of 5 % to 100 % of MeCN in H_2O (10mM Ammonium Formate, adjusted to pH 4 with Formic Acid). The %MeCN is kept stable at 5% from 0.00-0.50 minutes and gradually increased to 100% from 0.50-2.50 minutes. From 2.50-3.50 minutes, the %MeCN is kept stable at 100%. Method A50 is a 3.50 minute method using a Kinetex EVO C18 (5 μM, 4.6 x 50 mm) column, a flow rate of 2.20 mL/min, and a low pH buffer gradient of 5 % to 100 % of MeCN in H_2O (10mM Ammonium Formate, adjusted to pH 4 with Formic Acid). The %MeCN is kept stable at 50% from 0.00-0.50 minutes and gradually increased to 100% from 0.50-2.50 minutes. From 2.50-3.50 minutes, the %MeCN is kept stable at 100%. Method B05 is a 3.50 minute method using a Gemini NX-C18 (5 μM, 4.6 x 50 mm) column, a flow rate of 2.20 mL/min, and a high pH buffer gradient of 5 % to 100 % of MeCN in H_2O (10mM Ammonium Bicarbonate, adjusted to pH 10 with Ammonium Hydroxide). The %MeCN is kept stable at 5% from 0.00-0.50 minutes and gradually increased to 100% from 0.50-2.50 minutes. From 2.50-3.50 minutes, the %MeCN is kept stable at 100%. Method B50 is a 3.50 minute method using a Gemini NX-C18 (5 μM, 4.6 x 50 mm) column, a flow rate of 2.20 mL/min, and a high pH buffer gradient of 50 % to 100 % of MeCN in H_2O (10mM Ammonium Bicarbonate, adjusted to pH 10 with Ammonium Hydroxide). The %MeCN is kept stable at 50% from 0.00-0.50 minutes and gradually increased to 100% from 0.50-2.50 minutes. From 2.50-3.50 minutes, the %MeCN is kept stable at 100%.

The analytical QA/QC chromatograms were also performed using a Waters Acquity UPLC instrument with DAD detector (200 nm to 320 nm) and a thermostatted column compartment set to 35 ^oC. The mass spectra were recorded with a Waters SQD detector with the desolvation temperature set to 450 ^oC, the source temperature set to 150 ^oC and the desolvation gas flow set to 1000 L/hr. The mass spectrometer was equipped with an electrospray ion source (ESI) operated in a positive, or negative, ion mode and was set to scan between m/z 100-2000 with a scan time of 0.2s. Products were analyzed by UPLC/MS on one of 3 methods (10mM Ammonium Formate pH4/10mM Ammonium Bicarbonate pH10/0.1% TFA). All 3 methods are 10.00 minute methods

using an Acquity UPLC BEH C18 (1.7 µM, 2.1x50mm) column and a flow rate of 0.700 mL/min. The gradients are 5%-100% MeCN from 0.00-9.00 minutes and then kept stable at 100% MeCN from 9.00-10.00 minutes.

The ^{1}H NMR spectra were recorded on a Bruker Avance II 300 MHz instrument and a Bruker Avance DRX-500 MHz instrument. The chemical shifts are reported in parts-per-million and are referenced to solvent peaks, which in ^{1}H NMR appear at 7.26 ppm for $CDCl_3$, 2.50 for DMSO-d_6, and 3.31 ppm for CD_3OD.

Structure	Name	ID
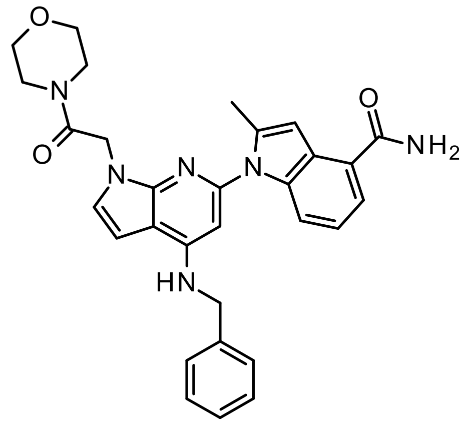	1-[4-(benzylamino)-1-[2-(morpholin-4-yl)-2-oxoethyl]-1*H*-pyrrolo[2,3-b]pyridin-6-yl]-2-methyl-1*H*-indole-4-carboxamide	**GND-028**
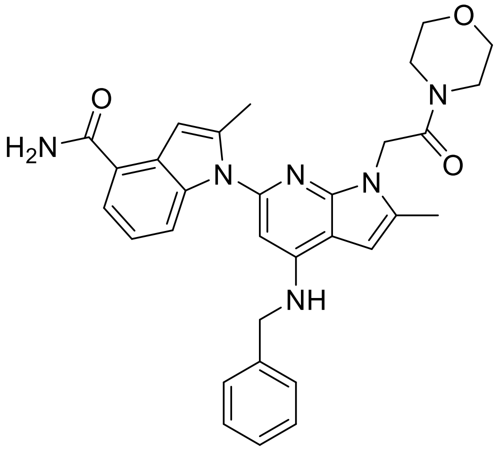	(1-[4-(Benzylamino)-2-methyl-1-[2-(morpholin-4-yl)-2-oxoethyl]-1*H*-pyrrolo[2,3-b]pyridin-6-yl]-2-methyl-1*H*-indole-4-carboxamide	**GND-135**

GND-028, Step 1: 2-[(4,6-dichloropyrrolo[2,3-b]pyridin-1-yl)methoxy]ethyl-trimethyl-silane

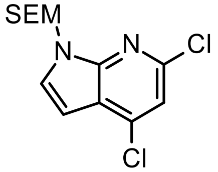

A solution of 4,6-dichloro-1*H*-pyrrolo[2,3-b]pyridine (10.0 g, 53.5 mmol) in anhydrous DMF (80.0 mL) was added to a suspension of NaH (60% oil dispersion, 3.42 g, 85.5 mmol) in anhydrous DMF (80.0 mL) at 0 °C. The mixture was stirred at 0 °C for 15 min, and 2-(chloromethoxy)ethyl-trimethyl-silane (10.7 g, 64.2 mmol) was added at 0 °C. The mixture was warmed to 23°C and stirred for 2 h. The mixture was cooled to 0 °C and diluted with a saturated aqueous solution of NH_4Cl (500 mL) and EtOAc (800 mL). The phases were separated, and the organic phase was washed with water (2 x 500 mL) and brine (2 x 250 mL), dried (Na_2SO_4), filtered, and concentrated. The residue was purified by silica gel chromatography (220 g cartridge) with hexanes and EtOAc (0-20%) to provide the title compound as an oil (14.92 g, 88% yield). ^1H NMR (300 MHz, $CDCl_3$) δ 7.36 (d, J = 3.6 Hz, 1H), 7.16 (d, J = 0.7 Hz, 1H), 6.67 – 6.56 (m, 1H), 5.62 (s, 2H), 3.61 – 3.42 (m, 2H), 1.00 – 0.81 (m, 2H), -0.05 (d, J = 0.8 Hz, 9H).

GND-028, Step 2: *N*-benzyl-6-chloro-1-(2-trimethylsilylethoxymethyl)pyrrolo[2,3-b]pyridin-4-amine

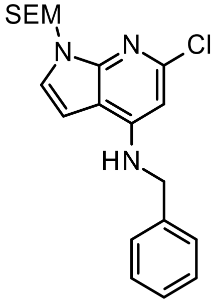

N-Ethyl-*N*-isopropyl-propan-2-amine (18.2 g, 141 mmol) was added to a mixture of 2-[(4,6-dichloropyrrolo[2,3-b]pyridin-1-yl)methoxy]ethyl-trimethyl-silane (14.9 g, 47.0 mmol) and benzylamine (10.1 g, 94.0 mmol) in NMP (30.0 mL). The mixture was stirred at 165 °C for 18 hr. The mixture was cooled to 23°C and diluted with an aqueous solution of HCl (1M, 100 mL). The aqueous phase was extracted with EtOAc (350 mL). The combined organic phases were washed with saturated aqueous $NaHCO_3$ (150 mL) and brine (150 mL), dried (Na_2SO_4), filtered, and concentrated. The residue was purified by silica gel chromatography (220 g cartridge) with hexanes and EtOAc (0-25%) to provide the title compound as a solid (11.3 g, 62%). ^1H NMR (300 MHz, $CDCl_3$) δ 7.49 – 7.30 (m, 5H), 7.10 (dd, J = 3.7, 0.9 Hz, 1H), 6.35 (dd, J = 3.7, 0.9 Hz, 1H), 6.29 (s, 1H), 5.57 (d, J = 0.9 Hz, 2H), 4.79 (d, J = 5.7 Hz, 1H), 4.50 (d, J = 5.5 Hz, 2H), 3.54 (td, J = 8.1, 0.9 Hz, 2H), 0.99 – 0.80 (m, 2H), -0.05 (d, J = 1.0 Hz, 9H). m/z: ES+ $[M+H]^+$ = 388.4; LCMS (A05); t_R = 3.03 min.

<u>GND-028, Step 3:</u> 1-[4-(benzylamino)-1-methyl-pyrrolo[2,3-b]pyridin-6-yl]-2-methyl-indole-4-carbonitrile

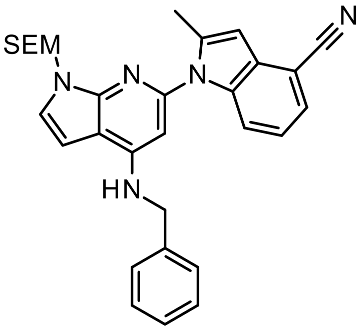

Degassed anhydrous toluene (200 mL) was added to a mixture of *N*-benzyl-6-chloro-1-(2-trimethylsilylethoxymethyl)pyrrolo[2,3-b]pyridin-4-amine (8.00 g, 20.6 mmol), 2-methyl-1*H*-indole-4-carbonitrile (75.0 %, 5.58 g, 26.8 mmol), *t*-BuONa (3963 mg, 41.2 mmol), BippyPhos (3134 mg, 6.19 mmol), and [(Cinnamyl)PdCl]$_2$ (1602 mg, 3.09 mmol). The mixture was stirred at 110 °C for 3 h, cooled to 23 °C, and filtered through Celite washing with EtOAc (100 mL). The filtrate was concentrated. The residue was purified by silica gel chromatography (120 g cartridge) using hexanes and EtOAc (0-20%) to provide the title compound as an oil (12.0 g, 53%, 46% pure). ^{1}H NMR (300 MHz, CDCl$_3$) δ 7.43 (d, J = 4.1 Hz, 2H), 7.24 (d, J = 7.8 Hz, 9H), 6.54 (d, J = 3.7 Hz, 1H), 5.62 (s, 2H), 5.04 (s, 2H), 3.59 – 3.48 (m, 2H), 2.40 (s, 3H), 1.43 (s, 9H), 0.88 (t, J = 8.2 Hz, 2H). m/z: ES+ $[M+H]^+$ 508.3 ; LCMS (A05); t_R = 2.85 m.

<u>GND-028, Step 4:</u> 1-[4-(benzylamino)-1-(2-trimethylsilylethoxymethyl)pyrrolo[2,3-b]pyridin-6-yl]-2-methyl-indole-4-carboxamide

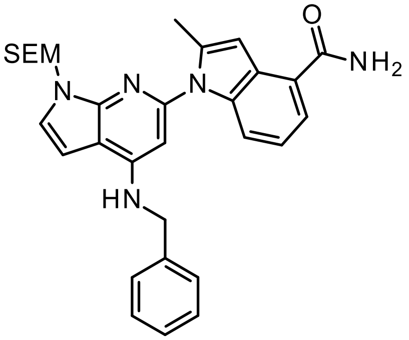

(1E)-Acetaldehyde oxime (3.21 g, 54.4 mmol) was added to a mixture of 1-[4-(benzylamino)-1-(2-trimethylsilylethoxymethyl)pyrrolo[2,3-b]pyridin-6-yl]-2-methyl-indole-4-carbonitrile (46.0 %, 12.0 g, 10.9 mmol), palladium acetate (0.488 g, 2.17 mmol), and triphenylphosphine (1.14 g, 4.35 mmol) in a mixture of EtOH/H_2O (8:1 v/v, 180 mL) at 23°C. The flask was sealed, and the mixture was stirred at 110 °C for 2 h. The mixture was cooled to 23°C and filtered through Celite washing with EtOAc (2 x 20.0 mL). The filtrate was concentrated, and the residue was purified by silica gel chromatography (220 g cartridge) with hexanes and ethyl acetate (0-100%) to provide the title compound as a solid (5.09 g, 89%). m/z: ES+ $[M+H]^+$ 526.2; LCMS (A05); t_R = 2.53 m.

GND-028, Step 5: 1-[4-(benzylamino)-1*H*-pyrrolo[2,3-b]pyridin-6-yl]-2-methyl-indole-4-carboxamide

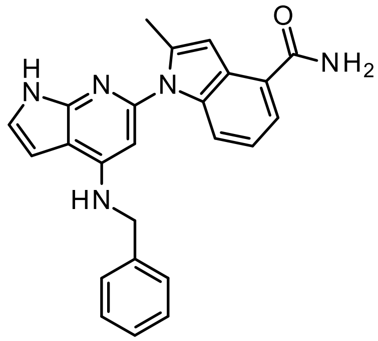

TFA (50.0 mL) was added to a solution of 1-[4-(benzylamino)-1-(2-trimethylsilylethoxymethyl)pyrrolo[2,3-b]pyridin-6-yl]-2-methyl-indole-4-carboxamide (5.09 g, 9.68 mmol) in DCM anhydrous (150 mL) at 0 °C. The mixture was stirred at 23°C for 3 h and concentrated. The residue was dissolved in 1,4-dioxane (150 mL), and a saturated aqueous solution of NH_4OH (50.0 mL) was added. The mixture was stirred at 60 °C for 16 h and concentrated. The residue was purified by silica gel chromatography (80 g cartridge) with ethyl acetate in hexanes (0-100%) to provide the title compound as a solid (3.09 g, 81%). ^{1}H NMR (300 MHz, DMSO) δ 11.39 (s, 1H), 7.68 (t, J = 6.0 Hz, 2H), 7.45 – 7.23 (m, 6H), 7.19 (t, J = 2.9 Hz, 2H), 6.99 – 6.83 (m, 2H), 6.80 (d, J = 1.3 Hz, 1H), 6.72 (dd, J = 3.5, 2.1 Hz, 1H), 6.01 (s, 1H), 4.52 (d, J = 6.0 Hz, 2H), 2.25 (s, 3H). m/z: ES+ $[M+H]^+$ 396.3 ; LCMS (A05); t_R = 2.36 m.

GND-028, Step 6: 2-[4-(benzylamino)-6-(4-carbamoyl-2-methyl-indol-1-yl)pyrrolo[2,3-b]pyridin-1-yl]acetic acid

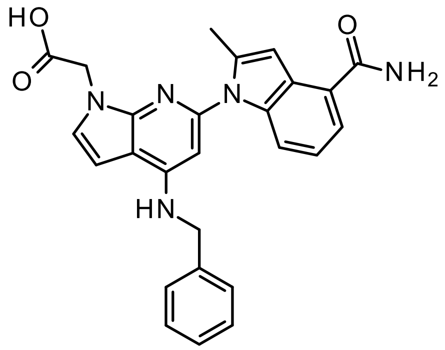

Sodium hydride (60.0 %, 26 mg, 0.68 mmol) was added to a solution of 1-[4-(benzylamino)-1*H*-pyrrolo[2,3-b]pyridin-6-yl]-2-methyl-indole-4-carboxamide (245 mg, 0.620 mmol) in dry DMF (5.00 mL) under N_2 at 23°C. After stirring for 30 min, a solution of *tert*-butyl 2-bromoacetate (133 mg, 0.681 mmol) in dry DMF (2.00 mL) was added. The mixture was stirred at 23°C for 1 h. Water (1.00 mL) was added to the mixture, and the solvent was concentrated. The residue was dissolved in DCM (3.00 mL), and TFA (3.00 mL) was added. After stirring the mixture for 15 h, the mixture was concentrated. The residue (500 mg) was taken to the next step without purification. m/z: ES+ $[M+H]^+$ 454.1. LCMS (A05) t_R = 2.05 min.

GND-028, Step 7: 1-[4-(benzylamino)-1-[2-(morpholin-4-yl)-2-oxoethyl]-1*H*-pyrrolo[2,3-b]pyridin-6-yl]-2-methyl-1*H*-indole-4-carboxamide

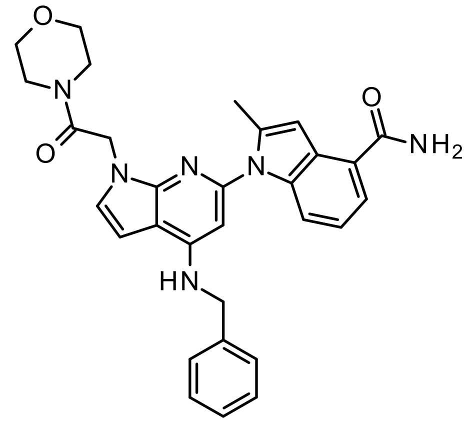

HATU (419.0 mg, 1.10 mmol) was added to a mixture of DIEA (0.377 mL, 2.21 mmol), 2-[4-(benzylamino)-6-(4-carbamoyl-2-methyl-indol-1-yl)pyrrolo[2,3-b]pyridin-1-yl]acetic acid (250 mg, 0.551 mmol) and morpholine (0.121 mL, 1.38 mmol) in dry DMF (5.00 mL) under N_2 at 23°C. The mixture was stirred at 23°C for 2 h. The mixture was concentrated, and the residue was purified using reverse phase HPLC (40 g, C18 column) with 10-80% ACN in Water (10mMol ammonium formate) as eluent to obtain the title product (230 mg, 80%) as a solid. ^{1}H NMR (500 MHz, DMSO) δ 7.75 (s, 1H), 7.68 (s, 1H), 7.42 (d, J = 7.3 Hz, 1H), 7.36 (dt, J = 14.9, 7.3 Hz, 4H), 7.28 (t, J = 7.0 Hz, 1H), 7.18 (d, J = 3.5 Hz, 2H), 6.96 (d, J = 8.1 Hz, 1H), 6.86 (t, J = 7.8 Hz, 1H), 6.81 (s, 1H), 6.73 (d, J = 3.4 Hz, 1H), 6.03 (s, 1H), 5.05 (s, 2H), 4.54 (d, J = 5.9 Hz, 2H), 3.54 (s, 6H), 3.41 (s, 2H), 2.24 (s, 3H). m/z: ES+ $[M+H]^+$ 523.8, LCMS (A05) t_R = 2.16 min.

GND-135, Step 1: 1-(Benzenesulfonyl)-4,6-dichloro-1*H*-pyrrolo[2,3-b]pyridine

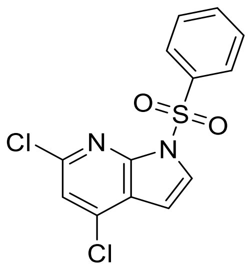

4,6-Dichloro-1h-pyrrolo[2,3-b]pyridine (20.0 g, 107 mmol) was dissolved in anhydrous DMF (320 mL), and NaH (60% oil dispersion, 6.42 g, 160 mmol) was added at 0 °C. The mixture was stirred at 0 °C for 15 min, and benzenesulfonyl chloride (16.4 mL, 128 mmol) was added. The mixture was slowly warned and stirred at 23°C for 16 hours. The mixture was cooled to 0 °C, and diluted with a saturated aqueous solution of NH_4Cl (100 mL), and the aqueous phase was extracted with DCM (2 x 100 mL). The combined organic phases were washed with water and brine, dried ($MgSO_4$), filtered, and concentrated. The residue was triturated with MeCN to provide the title compound as a solid (30.5 g, 87% yield). m/z (ES+) $[M+H]^+$ = 327; LCMS (A05); t_R = 2.84 min.

GND-135, Step 2: 1-(Benzenesulfonyl)-4,6-dichloro-2-methyl-1*H*-pyrrolo[2,3-b]pyridine

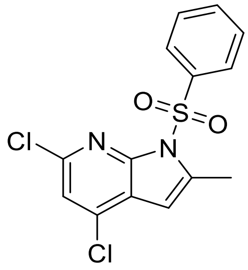

1-(Benzenesulfonyl)-4,6-dichloro-1*H*-pyrrolo[2,3-b]pyridine (5.00 g, 15.3 mmol) was dissolved in dry THF (75 mL) and added dropwise to a freshly prepared solution of LDA (prepared by the addition of *n*-BuLi (2.5 M in hexane, 12.2 mL, 30.6 mmol) to a solution of diisopropylamine (4.30 mL, 30.6 mmol) in dry THF (45 mL) followed by stirring for 30 min at -78°C). The mixture was warmed to 0 °C, and MeI (1.90 mL, 30.6 mmol) was added dropwise. The mixture was stirred for 3h at 0°C and 23°C for 16 hours. The mixture was diluted with a saturated aqueous solution of NH_4Cl (100 mL) and extracted with EtOAc (2 x 100 mL). The combined organic phases were washed with water and brine, dried ($MgSO_4$), filtered, and concentrated. The residue was purified by silica gel chromatography using 0-20% EtOAc in cyclohexane to provide the title compound as a solid (1.18 g 23% yield). m/z (ES+) $[M+H]^+$= 341; LCMS (A05); t_R = 1.23 min.

<u>GND-135, Step 3:</u> 1-(Benzenesulfonyl)-*N*-benzyl-6-chloro-2-methyl-1*H*-pyrrolo[2,3-b]pyridin-4-amine

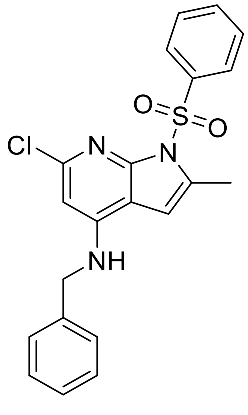

1-(Benzenesulfonyl)-4,6-dichloro-2-methyl-1*H*-pyrrolo[2,3-b]pyridine (1.0g, 2.93 mmol) was dissolved in NMP (9 mL), and DIPEA (1.53 mL, 8.79 mmol) and benzylamine (6, 961 uL, 8.79 mmol) were added. The mixture was stirred at 200 °C for 16 hours. The mixture was cooled to 23°C and diluted with water. The aqueous phase was extracted with cyclohexane (100 mL). The combined organic phase was dried ($MgSO_4$), filtered, and concentrated. The residue was purified by silica gel chromatography using 0-20% EtOAc in cyclohexane to provide the title compound as a solid (570 mg, 47% yield). *m/z* (ES+) MS $[M+H]^+$ = 412; LCMS (A05); t_R = 1.15 min.

<u>GND-135, Step 4:</u> 1-[1-(Benzenesulfonyl)-4-(benzylamino)-2-methyl-1*H*-pyrrolo[2,3-b]pyridin-6-yl]-2-methyl-1*H*-indole-4-carbonitrile

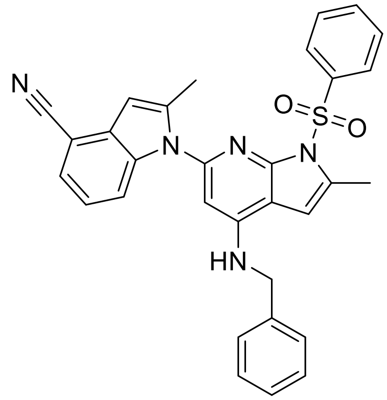

1-(Benzenesulfonyl)-*N*-benzyl-6-chloro-2-methyl-1*H*-pyrrolo[2,3-b]pyridin-4-amine (570 mg, 1.38 mmol) was dissolved in dry toluene (7 mL), and 2-Methyl-1*H*-indole-4-carbonitrile (216 mg, 1.38 mmol), *t*-BuONa (206 mg, 2.14 mmol), $[PdCl(cinnamyl)]_2$ (143 mg, 0.28 mmol), and BippyPhos (295 mg, 0.58 mmol) were added at 23°C under nitrogen. The mixture was stirred at 110 °C for 2 h. The mixture was cooled to 23°C and filtered through a short pad of Celite, washing with EtOAc. The filtrate was concentrated, and the residue was purified by silica gel chromatography using 0-25% EtOAc in cyclohexane to provide the title compound as a solid (191 mg, 26% yield). m/z (ES+) [M+H]+; = 532; LCMS (A05); t_R = 1.23 min.

<u>GND-135, Step 5:</u> 1-[1-(Benzenesulfonyl)-4-(benzylamino)-2-methyl-1H**-pyrrolo[2,3-b]pyridin-6-yl]-2-methyl-1*H*-indole-4-carboxamide**

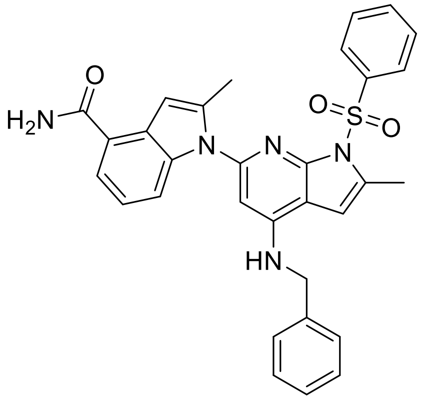

Acetaldoxime (108 mL, 1.80 mmol), 1-[1-(Benzenesulfonyl)-4-(benzylamino)-2-methyl-1*H*-pyrrolo[2,3-b]pyridin-6-yl]-2-methyl-1*H*-indole-4-carbonitrile (191 mg, 0.36 mmol), $Pd(OAc)_2$ (16.9 mg, 0.08 mmol), and TPP (22.6 mg, 0.09 mmol) were dissolved in a mixture of $EtOH/H_2O$ (8:1 v/v, 8 mL) at 23°C under nitrogen, and the flask was sealed. The mixture was stirred at 110 °C for 3 hours. The mixture was cooled to rt, filtered through a short pad of Celite, washing with EtOAc. The filtrate was concentrated, and the residue was purified by silica gel chromatography using 0-5% MeOH in DCM to provide the title compound as a solid (120 mg, 61%). m/z (ES+) $[M+H]^+$= 550; LCMS (A05); t_R = 0.99 min.

<u>GND-135, Step 6:</u> 1-[4-(Benzylamino)-2-methyl-1*H*-pyrrolo[2,3-b]pyridin-6-yl]-2-methyl-1*H*-indole-4-carboxamide

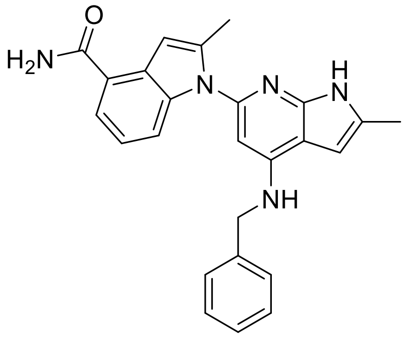

1-[1-(Benzenesulfonyl)-4-(benzylamino)-2-methyl-1*H*-pyrrolo[2,3-b]pyridin-6-yl]-2-methyl-1*H*-indole-4-carboxamide (300 mg (70% purity), 0.38 mmol) was dissolved in 1,4-dioxane (3 mL), and aqueous solution of NaOH (2M, 955 uL, 1.91 mmol) was added. The mixture was stirred at 80°C for 16 hours. The mixture was concentrated, and the residue was purified by prep HPLC to provide the title compound as a solid (87 mg, 55% yield). ^{1}H NMR (500 MHz, DMSO) δ 11.21 (s, 1H), 7.68 (s, 1H), 7.47 (t, J = 6.1 Hz, 1H), 7.40 (d, J = 7.2 Hz, 1H), 7.38 – 7.30 (m, 4H), 7.27 (t, J = 6.4 Hz, 1H), 7.18 (s, 1H), 6.92 (d, J = 8.1 Hz, 1H), 6.86 (t, J = 7.7 Hz, 1H), 6.78 (s, 1H), 6.37 (s, 1H), 5.96 (s, 1H), 4.49 (d, J = 6.0 Hz, 2H), 2.35 (s, 3H), 2.23 (s, 3H). m/z (ES+) $[M+H]^+$ 410; LCMS (A05); t_R = 4.67 min.

<u>GND-135, Step 7:</u> 1-[4-(Benzylamino)-2-methyl-1-[2-(morpholin-4-yl)-2-oxoethyl]-1*H*-pyrrolo[2,3-b]pyridin-6-yl]-2-methyl-1*H*-indole-4-carboxamide

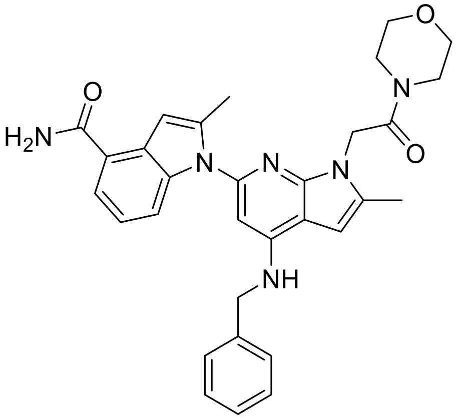

1-[4-(Benzylamino)-2-methyl-1*H*-pyrrolo[2,3-b]pyridin-6-yl]-2-methyl-1*H*-indole-4-carboxamide (40 mg, 0.10 mmol) was dissolved in dry DMF (0.8 mL), and NaH (60% oil dispersion 4.30 mg, 0.11 mmol) was added. The mixture was stirred at rt for 30 min. A solution of *N*-(chloroacetyl)morpholine (13.6 uL, 0.11 mmol) in dry DMF (0.4 mL) was added, and the mixture was stirred at 23°C for 1h. The mixture was concentrated, and the residue was purified by HPLC to provide the title compound as a solid (11 mg, 21% yield). ^{1}H NMR (500 MHz, DMSO) δ 7.68 (s, 1H), 7.59 (t, J = 6.1 Hz, 1H), 7.41 (d, J = 7.2 Hz, 1H), 7.35 (q, J = 7.5 Hz, 4H), 7.28 (t, J = 6.8 Hz, 1H), 7.18 (s, 1H), 6.91 (d, J = 8.1 Hz, 1H), 6.85 (t, J = 7.7 Hz, 1H), 6.79 (s, 1H), 6.47 (s, 1H), 6.00 (s, 1H), 5.01 (d, J = 9.0 Hz, 2H), 4.52 (d, J = 5.2 Hz, 2H), 3.56 (s, 4H), 3.50 (s, 2H), 3.41 (s, 2H), 2.31 (s, 3H), 2.23 (s, 3H). m/z (ES+) $[M+H]^+$ =537; LCMS (A05); t_R = 4.39 min.

GND-135

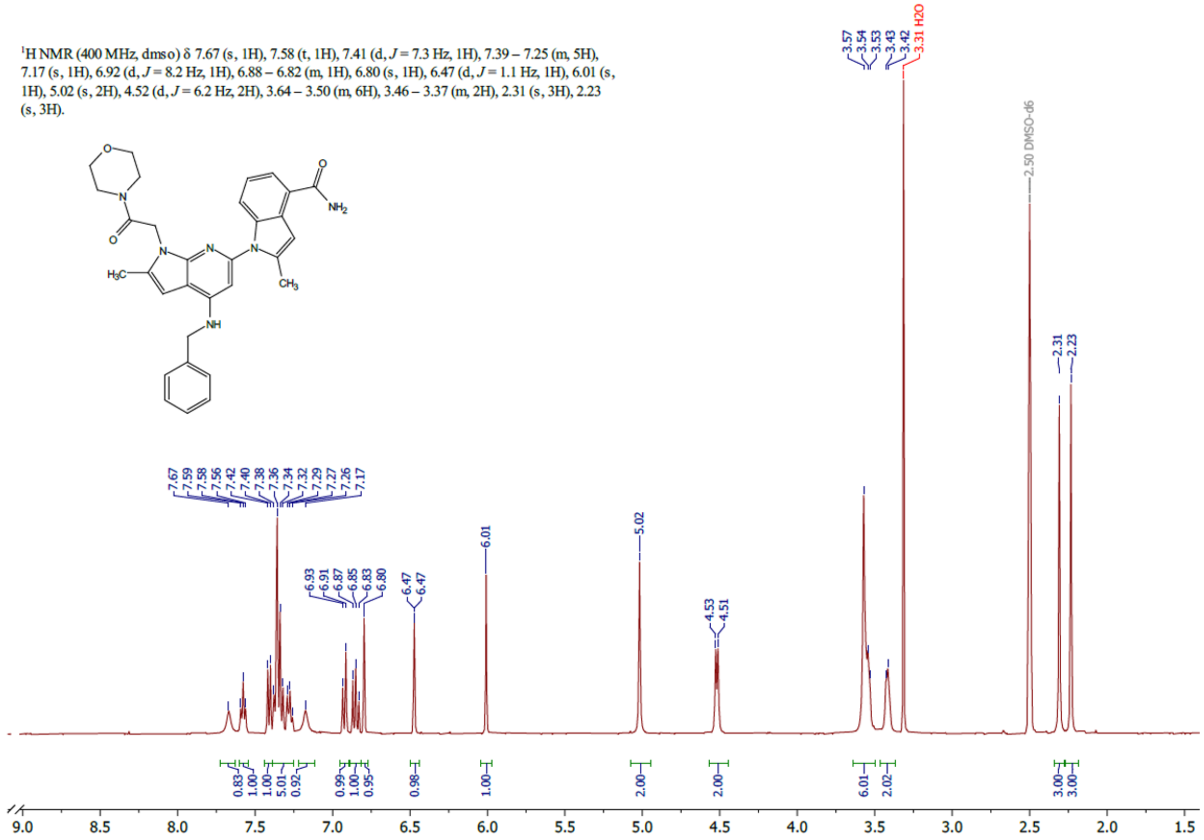
1H NMR (400 MHz, dmso) δ 7.67 (s, 1H), 7.58 (t, 1H), 7.41 (d, J = 7.3 Hz, 1H), 7.39 – 7.25 (m, 5H), 7.17 (s, 1H), 6.92 (d, J = 8.2 Hz, 1H), 6.88 – 6.82 (m, 1H), 6.80 (s, 1H), 6.47 (d, J = 1.1 Hz, 1H), 6.01 (s, 1H), 5.02 (s, 2H), 4.52 (d, J = 6.2 Hz, 2H), 3.64 – 3.50 (m, 6H), 3.46 – 3.37 (m, 2H), 2.31 (s, 3H), 2.23 (s, 3H).
O
N
O
N
N
N
N
H3C
NH
O
NH2
CH3
3.31 H2O
2.50 DMSO-d6
9.0
8.5
8.0
7.5
7.0
6.5
6.0
5.5
5.0
4.5
4.0
3.5
3.0
2.5
2.0
1.5

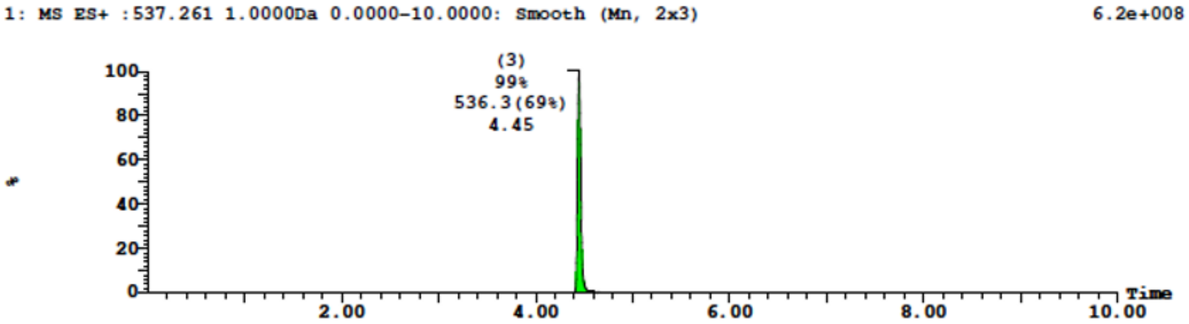
1: MS ES+ :537.261 1.0000Da 0.0000-10.0000: Smooth (Mn, 2x3)
6.2e+008
(3)
99%
536.3(69%)
4.45
%
Time
2.00
4.00
6.00
8.00
10.00

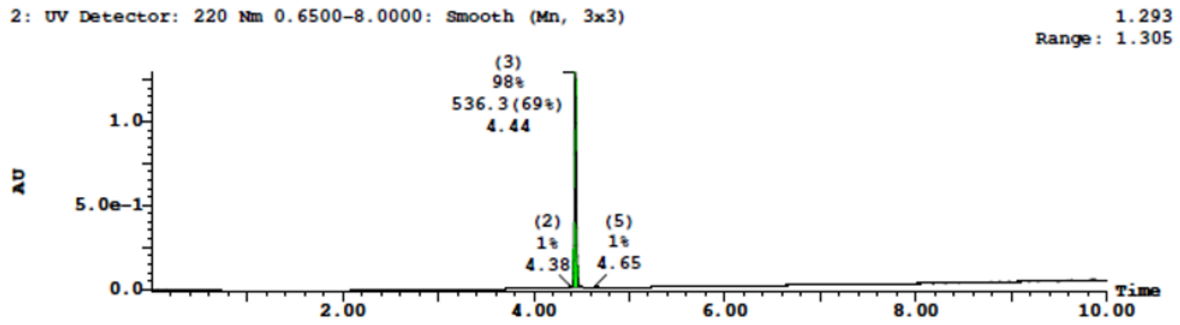

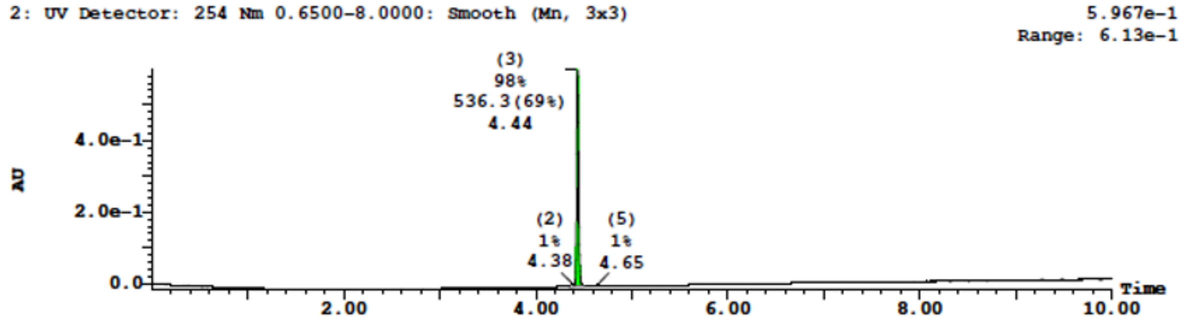

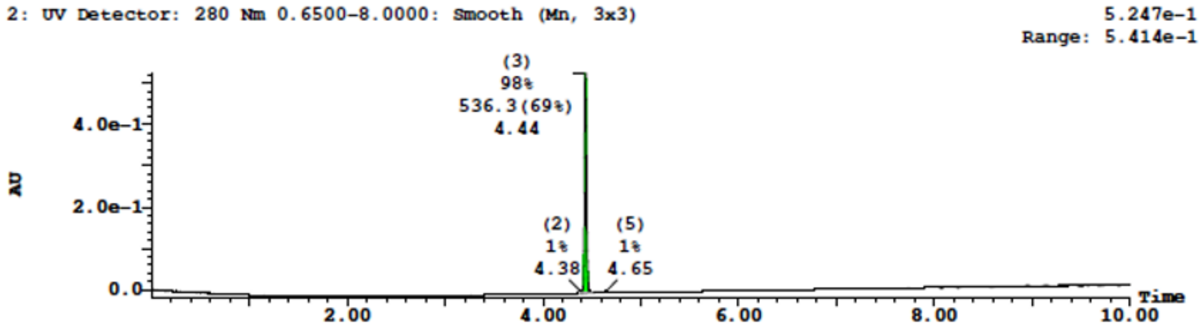

Supplementary References:

Zhou, H.-J., Wang, J., Yao, B., Wong, S., Djakovic, S., Kumar, B., Rice, J., Valle, E., Soriano, F., Menon, M.-K., Madriaga, A., Kiss von Soly, S., Kumar, A., Parlati, F., Yakes, F. M., Shawver, L., Le Moigne, R., Anderson, D. J., Rolfe, M. & Wustrow, D. (2015). *J Med Chem* 58, 9480–9497.
